# Análise de desempenho dos centros dialíticos no encaminhamento e inscrição em lista de espera pré-transplante renal em Minas Gerais, Brasil, 2015 a 2019

**DOI:** 10.1590/0102-311XPT021423

**Published:** 2025-01-27

**Authors:** Cláudio Vitorino Pereira, Isabel Cristina Gonçalves Leite, Mário Círio Nogueira, Gustavo Fernandes Ferreira

**Affiliations:** 1 Universidade Federal de Juiz de Fora, Juiz de Fora, Brasil.; 2 Santa Casa de Misericórdia de Juiz de Fora, Juiz de Fora, Brasil.

**Keywords:** Transplante de Rim, Disparidades em Assistência à Saúde, Encaminhamento e Consulta, Kidney Transplantation, Healthcare Disparities, Referral and Consultation, Trasplante de Riñón, Disparidades en Atención de Salud, Derivación y Consulta

## Abstract

Apesar da relevância do transplante renal, a oferta de órgãos e o processo para inclusão na lista de espera ainda representam entraves. O presente estudo teve como objetivo analisar o desempenho dos centros dialíticos no encaminhamento para avaliação pré-transplante renal e inclusão em lista de espera de pacientes incidentes em diálise, no período de 2015 a 2019, no Estado de Minas Gerais, Brasil. Trata-se de um estudo de coorte retrospectivo, que teve como amostra 23.297 registros de pacientes que realizaram terapia dialítica em instituições públicas, filantrópicas ou que tiveram seu tratamento custeado pelo Sistema Único de Saúde em clínicas privadas conveniadas. Para avaliação do encaminhamento pré-transplante renal, realizou-se a análise de sobrevida pelo método Kaplan-Meier. A força da associação entre as variáveis de exposição e a ocorrência da inscrição na lista foram analisadas por meio dos modelos de regressão de Cox. Ao analisar a inscrição em lista de espera pré-transplante renal, 14,8% da amostra encontrava-se inscrita. A probabilidade acumulada de inscrição foi de 1,2% em 180 dias e 3,3% em um ano, entre 2016 e 2019. A macrorregião Norte apresentou maior probabilidade acumulada de inscrição em lista de espera, enquanto a Sul apresentou a menor. Assim como indivíduos adultos apresentaram maior probabilidade de inscrição em relação aos idosos, e o ano de 2016 obteve maior probabilidade em relação aos anos de 2018 e 2019. Embora estabelecido na *Portaria nº 389/2014* do Ministério da Saúde o percentual mínimo de pacientes aptos que devem ser inseridos em lista, não foi observada repercussão positiva na probabilidade acumulada de inscrição entre os incidentes em diálise nos anos subsequentes.

## Abstract (en)

Despite the relevance of kidney transplantation, the supply of organs and the process for inclusion in its waiting list still represent obstacles. This study aimed to analyze the performance of dialysis centers in referring patients for pre-kidney transplant evaluation and inclusion in the waiting list of incident dialysis patients from 2015 to 2019 in the state of Minas Gerais, Brazil. This retrospective cohort study sampled 23,297 records of patients who underwent dialysis therapy in public or philanthropic institutions or who had their treatment funded by the Brazilian Unified National Health System in private clinics. Survival analysis by the Kaplan-Meier method was used to evaluate pre-kidney transplant referral. The strength of the association between the exposure variables and the occurrence of inclusion on the list were analyzed by Cox regression models. Only 14.8% of the sample was enrolled on the pre-kidney transplant waiting list. The cumulative probability of enrollment totaled 1.2% in 180 days and 3.3% in one year from 2016 to 2019. Northern Minas Gerais had the highest cumulative probability of enrollment on the waiting list, whereas its South, the lowest. Adult individuals were more likely to be enrolled than older adults, and enrollment was more likely in 2016 than in 2018 and 2019. Although *Ordinance n. 389/2014* from the Brazilian Ministry of Health established the minimum percentage of eligible patients that must be included in the list, this study observed no positive repercussion on the accumulated probability of enrollment in dialysis incidents in subsequent years.

## Resumen

A pesar de la relevancia del trasplante renal, el suministro de órganos y el proceso de inclusión en la lista de espera siguen representando obstáculos. Este estudio tuvo como objetivo analizar el desempeño de los centros de diálisis en la derivación para evaluación renal previa al trasplante e inclusión en la lista de espera de pacientes incidentes en diálisis en el período de 2015 a 2019, en el estado de Minas Gerais, Brasil. Se trata de un estudio de cohorte retrospectivo con una muestra de 23.297 registros de pacientes que se sometieron a terapia de diálisis en instituciones públicas, filantrópicas o que tuvieron su tratamiento financiado por el Sistema Único de Salud en clínicas afiliadas privadas. Para evaluar la derivación previa al trasplante de riñón, se realizó un análisis de supervivencia utilizando el método de Kaplan-Meier. La fuerza de la asociación entre las variables de exposición y la ocurrencia de inscripción en la lista se analizaron utilizando modelos de regresión de Cox. Se encontró que un 14,8% de la muestra estaba inscrita en la lista de espera previa al trasplante de riñón. La probabilidad acumulada de inscripción fue del 1,2% en 180 días y del 3,3% en un año, entre 2016 y 2019. La macrorregión Norte presentó la mayor probabilidad acumulada de inscripción en lista de espera, mientras que la Sur presentó la más baja. Además, los individuos adultos tuvieron una mayor probabilidad de inscribirse en comparación con los ancianos, y 2016 tuvo una mayor probabilidad en comparación con 2018 y 2019. Aunque está determinado en la *Ordenanza nº 389/2014* del Ministerio de Salud un porcentaje mínimo de pacientes elegibles que deben incluirse en la lista, no se encontró un impacto positivo en la probabilidad acumulada de inscripción en los incidentes de diálisis en los años posteriores.

## Introdução

O transplante renal constitui a proposta terapêutica ideal para a maioria dos pacientes com doença renal crônica (DRC) terminal, quando comparado às terapias dialíticas, pois melhora a qualidade de vida, reduz mortalidade, oferece expectativa de vida superior, apresenta menores taxas de hospitalização e substancial redução de custos do tratamento ^1^. Porém, a oferta de órgãos e o processo para inclusão na lista de espera ainda representam entraves ^2^.

A heterogeneidade na distribuição dos serviços dialíticos, a nível mundial, apresenta-se como um desafio para a garantia da equidade ao tratamento nefrológico ^3^. No Brasil, as regiões Sul e Sudeste concentram 65% dos centros de diálise, e aproximadamente 48,3% dos profissionais médicos especialistas em nefrologia encontram-se na Região Sudeste ^4^. Tal disparidade pode se refletir no diagnóstico, acompanhamento e tratamento da população com DRC e possibilidade de realização do transplante renal ^5^.

Segundo estimativas da Sociedade Brasileira de Nefrologia (SBN), havia, em 2021, 148.363 pessoas em tratamento dialítico no país ^4^. Destas, apenas 18,6% estavam com cadastro ativo na lista de espera. O Estado de Minas Gerais apresentava 20.314 pessoas em terapia dialítica e apenas 14,3% dos pacientes estavam cadastrados com possibilidade de receber o transplante renal ^6^.

O encaminhamento dos pacientes em diálise para avaliação em um centro transplantador é essencial para serem considerados candidatos ao transplante renal. Se aptos, são incluídos na lista de espera, com possibilidade de receber um órgão de acordo com critérios imunológicos de compatibilidade ^3^.

Na literatura científica, há dados escassos sobre o encaminhamento para avaliação pré-transplante renal e cadastro na lista de espera, o que dificulta o entendimento dessa etapa crucial para realização do transplante precoce ^3^.

Embora haja grande variação no percentual de transplantes renais realizados, o impacto exercido pelos centros dialíticos, assim como influência da heterogeneidade regional relacionada aos recursos de saúde, necessita de maior investigação, devido aos possíveis impactos relacionados ao acesso aos serviços de saúde ^7^. Por isso, há clara necessidade de monitorar o encaminhamento para transplante renal e avaliar as diferenças regionais, a fim de compreender as atuais barreiras para inclusão na lista de espera para transplante renal, tendo em vista garantir equidade ao acesso em todas as etapas das terapias renais substitutivas.

O presente estudo teve como objetivo analisar o desempenho dos centros dialíticos no encaminhamento para avaliação pré-transplante renal e inclusão em lista de espera de pacientes incidentes em diálise, no período de 2015 a 2019, no Estado de Minas Gerais.

## Metodologia

Trata-se de um estudo de coorte retrospectivo, que teve como população pacientes que realizaram terapia dialítica em instituições públicas, filantrópicas ou que tiveram seu tratamento custeado pelo Sistema Único de Saúde (SUS) em clínicas privadas conveniadas, no Estado de Minas Gerais.

Minas Gerais está localizado na Região Sudeste do Brasil, tem a quarta maior extensão territorial e destaca-se por ser o estado brasileiro com a maior quantidade de municípios, com 853 cidades, caracterizando-se por iniquidades socioeconômicas entre suas regiões ^8^.

A coleta de dados ocorreu por meio de análise do *status* em lista de espera para transplante renal cadastrado no Sistema Nacional de Transplante (SNT) e da Autorização para Procedimento de Alto Custo (APAC), ambos fornecidos pelo SNT em planilhas no programa Excel (https://products.office.com/). Não havia nos arquivos variáveis que permitissem a avaliação de desfechos clínicos.

Excluiu-se os registros que estavam em duplicidade ou que apresentavam local de residência fora do Estado de Minas Gerais. Foram incluídos na amostra registros de pacientes com idade igual ou superior a 18 anos, incidentes em diálise no período de janeiro de 2015 a dezembro de 2019 e em tratamento dialítico crônico, caracterizado por pelo menos 90 dias em diálise. A data da incidência em diálise foi determinada pela primeira sessão de hemodiálise ambulatorial ou com o início de diálise peritoneal domiciliar, definida por meio da geração da primeira APAC. O encaminhamento pré-transplante renal foi definido com a data da inclusão do paciente na lista de espera pelo centro transplantador, verificado nos registros do SNT. A identificação do paciente foi realizada pelo número identificador único, que permitiu a localização e o cruzamento das planilhas para unificação do banco de dados realizado no programa SPSS, versão 20 (https://www.ibm.com/).

Foram verificados fatores associados à inscrição na lista de espera para o transplante renal e, entre esses, os que foram submetidos ao transplante. Para análise da associação entre as variáveis, foi aplicado o teste qui-quadrado de Pearson.

Para avaliação do desempenho dos centros dialíticos, considerou-se o indicador de qualidade estabelecido na *Portaria nº 389/2014* do Ministério da Saúde ^9^, que determina que 80% dos pacientes aptos para transplante renal e com pelo menos seis meses em terapia dialítica devem estar inscritos na lista de espera do transplante renal, a partir do ano de 2016, que determina o marco do final do período de adaptação após a promulgação da referida recomendação.

Para avaliação do encaminhamento pré-transplante renal, realizou-se a análise de sobrevida pelo método Kaplan-Meier, no período de 2016 a 2019, com período de 365 dias de seguimento. O desfecho considerado foi o tempo decorrido até a inscrição na lista de espera para o transplante. As inscrições que ocorreram até o final do período de seguimento foram consideradas como eventos, e os demais pacientes foram censurados no tempo final de acompanhamento. Foram estimadas as probabilidades acumuladas de inscrição (complemento da probabilidade de sobrevida), com intervalo de 95% de confiança (IC95%), até 180 e 365 dias a partir da data inicial da diálise, estratificadas pelas variáveis sexo, faixa etária, natureza do serviço de diálise, macrorregião de saúde de residência e ano inicial de diálise.

Para avaliar a força da associação entre as variáveis de exposição e a ocorrência da inscrição na lista, ajustada pelas demais variáveis, foram feitos modelos de regressão de Cox, com estimação dos *hazard ratios* (HR) e IC95%. Com o objetivo de levar em conta a correlação entre os pacientes que residem em uma mesma região e, portanto, utilizam as mesmas redes de saúde, foi incluído no modelo de Cox um efeito aleatório por macrorregião de saúde, chamado modelo de Cox com fragilidade, com distribuição gama. O pressuposto de proporcionalidade dos riscos foi avaliado pela análise dos resíduos de Schoenfeld, por meio da análise gráfica e teste de hipóteses. Todas as análises de sobrevida foram feitas no programa R, versão 4.2.2 (http://www.r-project.org), com o pacote *survival*. Os resultados estatísticos obtidos nos testes foram considerados significativos quando p < 0,05.

## Resultados

Foram analisados 23.297 registros de pacientes que realizaram tratamento dialítico, no período de janeiro de 2015 a dezembro de 2019. Destes, 57% eram do sexo masculino e 55,1% eram de indivíduos com idade inferior a 60 anos. Ao analisar a inscrição em lista de espera pré-transplante renal, 14,8% da amostra encontrava-se inscrita no período de seguimento. A mediana da idade dos pacientes inscritos na lista de espera para o transplante renal foi 48 anos, enquanto a dos não inscritos foi de 61 anos.

A caracterização da amostra de acordo com o status na lista de espera e a realização do transplante renal são apresentados na Tabela 1. Em relação ao sexo, o maior percentual de inscritos na lista de espera pré-transplante renal e de transplantes renais realizados são referentes ao sexo masculino.

Indivíduos com idade inferior a 60 anos apresentaram maior percentual de inscrição na lista de espera pré-transplante e foram contemplados com maior percentual de transplantes renais, em relação aos idosos (Tabela 1).

**Tabela 1:** Caracterização da amostra segundo o *status* da lista de espera e transplante renal. Minas Gerais, Brasil, 2015 a 2019.

Variáveis	Pacientes	Inscritos	Valor de p	Transplantados	Valor de p *
	n (%)	n (%)		n (%)	
Sexo			0,002		< 0,001
Masculino	13.289 (57,0)	2.048 (15,4)		760 (5,7)	
Feminino	10.008 (43,0)	1.394 (13,9)		451 (4,5)	
Faixa etária			< 0,001		< 0,001
Não idoso (< 60 anos)	12.843 (55,1)	2.979 (23,2)		1.117 (8,7)	
Idoso (≥ 60 anos)	10.454 (44,9)	463 (4,4)		94 (0,9)	
Natureza da clínica			< 0,001		0,007
Privada	18.480 (79,3)	2.856 (15,5)		976 (5,3)	
Pública	2.843 (12,2)	358 (12,6)		148 (5,2)	
Filantrópica	1.281 (5,5)	119 (9,3)		42 (3,3)	
Universitária	693 (3,0)	109 (15,7)		45 (6,5)	
Macrorregião			< 0,001		< 0,001
Centro	8.113 (34,8)	1.579 (19,5)		520 (6,4)	
Centro Sul	820 (3,5)	123 (15,0)		38 (4,6)	
Jequitinhonha	214 (0,9)	36 (1,0)		19 (8,9)	
Leste	597 (2,6)	73 (2,1)		30 (5,0)	
Leste do Sul	748 (3,2)	127 (3,7)		47 (6,3)	
Nordeste	689 (3,0)	71 (2,1)		28 (4,1)	
Noroeste	557 (2,4)	97 (2,8)		29 (5,2)	
Norte	1.536 (6,6)	328 (21,4)		111 (7,2)	
Oeste	1.257 (5,4)	152 (12,1)		59 (4,7)	
Sudeste	2.100 (9,0)	247 (11,8)		106 (5,0)	
Sul	3.805 (16,3)	189 (5,0)		105 (2,8)	
Triângulo do Norte	1.222 (5,2)	236 (19,3)		61 (5,0)	
Triângulo do Sul	719 (3,1)	64 (8,9)		22 (3,1)	
Vale do Aço	920 (3,9)	120 (13,0)		36 (3,9)	
Ano de início da diálise			< 0,001		< 0,001
2015	10.265 (44,1)	2.682 (26,1)		884 (8,6)	
2016	3.294 (14,1)	309 (9,4)		131 (4,0)	
2017	3.380 (14,5)	215 (6,4)		99 (2,9)	
2018	3.483 (15,0)	160 (4,6)		69 (2,0)	
2019	2.875 (12,3)	76 (2,6)		28 (1,0)	

* Teste qui-quadrado de Pearson.

No que se refere à natureza jurídica das clínicas, as clínicas privadas e universitárias apresentaram maior percentual de pacientes inscritos em lista de espera. Quando analisada a realização do transplante renal, as clínicas universitárias apresentaram maior percentual de pacientes transplantados (Tabela 1).

Quanto à regionalização, a macrorregião Centro apresentou o maior quantitativo dos pacientes em tratamento dialítico no período com 34,8%. Já as macrorregiões Jequitinhonha e Noroeste apresentaram os menores percentuais de pacientes em terapia dialítica com 0,9% e 2,4%, respectivamente. Em relação à inscrição em lista de espera, as macrorregiões Norte, com 21,4%, e Centro, com 19,5%, alcançaram os maiores percentuais. Já as macrorregiões Jequitinhonha, com 1%, Leste, 2,1%, e Nordeste 2,1% apresentaram o menor percentual de pacientes cadastrados na lista pré-transplante renal (Tabela 1).

Em relação aos maiores percentuais de transplantes renais realizados por macrorregião, Jequitinhonha apresentou uma proporção de 8,9% de seus pacientes e a Norte 7,2%. Já Sul e Triângulo do Sul obtiveram as menores proporções com 2,8% e 3,1% (Tabela 1).

**Figura 1:**
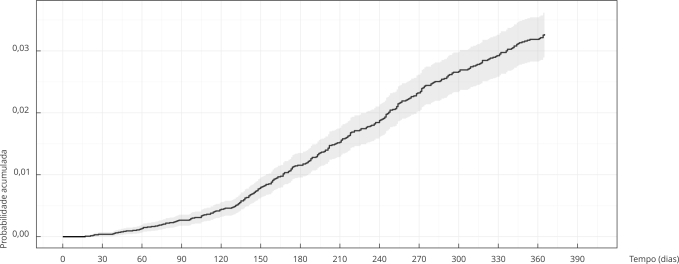
Probabilidade acumulada de inscrição na lista de espera para o transplante renal até um ano de seguimento. Minas Gerais, Brasil, 2016 a 2019.

**Figura 2:**
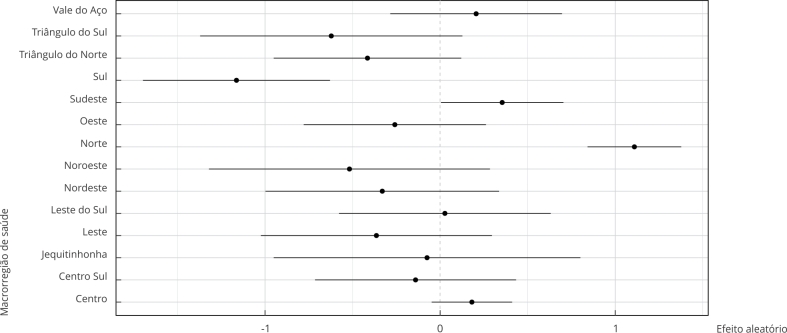
Efeitos aleatórios das macrorregiões de saúde no modelo de regressão de Cox com fragilidade, para o desfecho da inscrição em lista de espera para o transplante renal. Minas Gerais, Brasil, 2016 a 2019.

A Tabela 2 e as Figuras 1 e 2 apresentam as probabilidades acumuladas de inscrição na lista de transplante em 180 e 365 dias. A probabilidade acumulada de inscrição foi de 1,2% em 180 dias e 3,3% em um ano. Os indivíduos com idade inferior a 60 anos tiveram maior probabilidade acumulada de inscrição, mas não houve diferenças por sexo ou natureza do serviço. Houve grandes diferenças entre as macrorregiões de saúde, com destaque positivo para a Norte com 9,1% e negativo para a Sul com 0,8%. A probabilidade de inscrição foi menor para os anos de 2018 e 2019 em relação ao ano de 2016 (Tabela 2; Figura 2).

**Tabela 2:** Probabilidades acumuladas de inscrição na lista de transplante em 180 e 365 dias. Minas Gerais, Brasil, 2016 a 2019.

Variável	n	Probabilidade de inscrição [% (IC95%)]		Valor de p *
		180 dias	365 dias	
Geral	12.775	1,20 (1,00-1,40)	3,30 (2,90-3,60)	
Sexo				0,100
Feminino	5.515	0,96 (0,69-1,24)	2,92 (2,40-3,44)	
Masculino	7.260	1,30 (1,02-1,58)	3,54 (3,03-4,04)	
Faixa etária				< 0,001
Não idoso (< 60 anos)	6.263	2,11 (1,74-2,48)	5,76 (5,10-6,42)	
Idoso (≥ 60 anos)	6.512	0,18 (0,07-0,28)	0,55 (0,33-0,77)	
Natureza da clínica				0,200
Privada	9.936	1,20 (0,97-1,43)	3,40 (2,98-3,81)	
Pública	1.584	0,77 (0,32-1,23)	2,71 (1,79-3,63)	
Filantrópica	848	0,73 (0,00-1,45)	2,06 (0,68-3,42)	
Universitária	407	2,20 (0,68-3,70)	4,11 (1,86-6,30)	
Macrorregião				< 0,001
Centro	3.769	1,45 (1,05-1,85)	3,84 (3,12-4,55)	
Centro Sul	468	1,20 (0,14-2,25)	2,79 (1,05-4,51)	
Jequitinhonha	123	0,00 (0,00-0,00)	3,68 (0,00-7,70)	
Leste	386	0,57 (0,00-1,35)	2,34 (0,60-4,06)	
Leste do Sul	378	1,40 (0,17-2,61)	2,77 (0,95-4,55)	
Nordeste	470	0,00 (0,00-0,00)	2,65 (0,69-4,57)	
Noroeste	352	0,67 (0,00-1,60)	1,55 (0,02-3,06)	
Norte	880	4,54 (3,08-5,98)	9,10 (7,00-11,30)	
Oeste	794	0,77 (0,09-1,44)	2,32 (1,05-3,57)	
Sudeste	1.312	1,35 (0,61-2,07)	4,57 (3,10-6,01)	
Sul	2.081	0,23 (0,00-0,45)	0,81 (0,35-1,27)	
Triângulo do Norte	733	0,30 (0,00-0,70)	2,14 (0,92-3,33)	
Triângulo do Sul	464	0,24 (0,00-0,72)	1,53 (0,18-2,86)	
Vale do Aço	565	0,97 (0,12-1,81)	3,41 (1,68-5,11)	
Ano de início da diálise				< 0,001
2016	3.207	1,75 (1,27-2,23)	4,29 (3,51-5,06)	
2017	3.313	1,21 (0,82-1,60)	3,77 (3,05-4,49)	
2018	3.423	1,04 (0,68-1,40)	2,51 (1,93-3,08)	
2019	2.832	0,45 (0,17-0,74)	1,79 (0,97-2,60)	

IC95%: intervalo de 95% de confiança.

* Teste de *log-rank*.

Nos modelos de regressão de Cox, a inscrição na lista de espera manteve sua associação com a faixa etária do indivíduo, com pessoas idosas com menor probabilidade de inscrição, e os anos de 2018 e 2019 apresentaram menor probabilidade de ocorrer inscrição em relação a 2016 (Tabela 3). A respeito dos efeitos aleatórios, a única macrorregião de saúde com associação positiva foi a Norte, apresentando tempo menor até a inscrição, enquanto a Sul teve associação negativa com a probabilidade de inscrição na lista (Figura 2). O modelo de Cox cumpriu o pressuposto de proporcionalidade do risco.

**Tabela 3:** Resultados dos modelos de regressão simples e do modelo de regressão múltipla de Cox com fragilidade gama. Minas Gerais, Brasil, 2016 a 2019 (N = 12.775 pacientes *).

Variável	Modelos simples		Modelo múltiplo	
	HR	IC95%	HR	IC95%
Efeitos fixos				
Sexo				
Feminino	Referência		Referência	
Masculino	1,21	0,93-1,51	1,22	0,97-1,54
Faixa etária				
Não idoso (< 60 anos)	Referência		Referência	
Idoso (≥ 60 anos)	0,09	0,06-0,14	0,09	0,06-0,14
Natureza da clínica				
Privada	Referência		Referência	
Pública	0,79	0,55-1,14	0,78	0,53-1,13
Filantrópica	0,57	0,30-1,12	0,66	0,33-1,30
Universitária	1,22	0,70-2,13	1,20	0,67-2,16
Ano de início da diálise				
2016	Referência		Referência	
2017	0,87	0,66-1,13	0,96	0,73-1,25
2018	0,58	0,43-0,78	0,60	0,44-0,81
2019	0,39	0,25-0,61	0,39	0,25-0,61

HR: *hazard ratios*; IC95%: intervalo de 95% de confiança.

* Sem dados faltantes.

## Discussão

O percentual de pacientes em lista de espera para o transplante renal pode ser influenciado pela prevalência da DRC, mortalidade, critérios para inclusão na lista, competência transplantadora para captação e distribuição de órgãos, além de barreiras socioeconômicas e regionais ^7^. Percebe-se que apesar de haver um indicador de qualidade estabelecido na *Portaria nº 389/2014*, que determina o percentual mínimo de 80% dos pacientes aptos para transplante renal e com pelo menos seis meses em terapia dialítica estejam inscritos em lista de espera, com 14,8% dos participantes inscritos, o Estado de Minas Gerais apresenta desempenho distante do desejável.

Quando analisada a probabilidade acumulada de inscrição em lista de espera, os resultados são ainda mais preocupantes, pois o valor encontrado foi de apenas 3,3% ao ano, no período analisado. Destaca-se ainda um decréscimo na probabilidade de inscrição em lista de espera nos anos 2018 e 2019, efeito indesejado, devido ao término do prazo de readequação à diretriz do Ministério da Saúde no ano de 2016 ^9^.

Indivíduos do sexo feminino apresentaram menor percentual de inscrição em lista de espera. Outros estudos encontraram acesso desigual no encaminhamento e probabilidade de inscrição em lista de espera para transplante, nos quais as mulheres apresentaram piores resultados ^10^ ^,^ ^11^ ^,^ ^12^. Apesar de ser um processo potencialmente modificável, esses achados têm sido atribuídos à fragilidade do sexo feminino ^11^ ^,^ ^12^, que pode ser compreendida como estado de vulnerabilidade para desenvolver maior dependência ou mortalidade resultante de comorbidades ou condições de saúde ^13^.

Em relação à variável idade, os indivíduos idosos apresentaram menor percentual e probabilidade de inscrição em lista de espera e realização do transplante. A mediana da idade dos inscritos foi consideravelmente menor do que entre os não inscritos, logo a menor faixa etária pode ser compreendida como fator positivo associado ao desfecho inscrição. A idade elevada tem sido associada à maior probabilidade de remoção da lista de espera e à menor possibilidade de obtenção do transplante renal. Essa associação negativa fica mais evidente quanto mais avançada a idade dos indivíduos ^14^. O transplante renal em idosos tem propiciado melhor qualidade de vida e maior sobrevida para essa parcela populacional quando comparado às terapias dialíticas, porém devido às comorbidades e à possibilidade de complicações em decorrência da cirurgia e período pós-operatório, deve-se realizar uma avaliação criteriosa e individualizada de cada indivíduo ^15^.

Destaca-se que as clínicas de diálise devem ser responsáveis por orquestrar o acesso durante a terapia renal substitutiva, contudo o desempenho no encaminhamento e inserção em lista de espera tem sido muito variável ^3^. Estudos realizados no Estado da Geórgia (Estados Unidos) demostraram que apesar da complexidade de análise devido ao grande de número de instalações de diálise, os centros que realizaram intervenções de capacitação junto às equipes assistencial e educacionais direcionadas a pacientes e familiares conseguiram otimizar o desempenho no encaminhamento ao transplante renal, minimizando, assim, inequidades ^16^ ^,^ ^17^.

Ao analisarmos o desempenho macrorregional dos centros dialíticos, foram observadas disparidades em relação ao percentual de indivíduos em terapia renal substitutiva, inscritos em lista de espera e no percentual relativo de transplantes renais realizados. Destaca-se ainda que a macrorregião de saúde Norte obteve associação positiva para probabilidade de inscrição em lista de espera, enquanto a Sul teve associação negativa. Estudo que analisou o fluxo assistencial de atenção aos grandes queimados nas macrorregiões de saúde no Estado de Minas Gerais também destacou a macrorregião Norte como melhor exemplo de rede assistencial em alta complexidade para assistência de médio e grande queimados, devido a sua organização e capacidade para orquestrar a rede de urgência e emergência, assim como por ter limites bem determinados em seu território e sem sobrecarga de outras macrorregiões. Entende-se como grande queimado pacientes cuja extensão, profundidade e/ou localidade da queimadura requer assistência em centros especializados. Já a macrorregião Sul, no mesmo estudo, foi uma das regiões que não apresentou rede assistencial bem definida e tinha grande dependência de outras macrorregiões ^18^.

A heterogeneidade regional é de fundamental importância para analisarmos o acesso da população aos serviços de saúde, pois poderá impactar na taxa de transplante renal realizado por meio da captação e distribuição de órgãos, inclusão em lista de espera, carga de doença populacional, fatores socioeconômicos e no deslocamento ao centro dialítico ^7^. Para minimizar os entraves relacionados a fatores geográficos e socioeconômicos, torna-se necessário melhorias nos aspectos organizativos da rede assistencial, atrelado ao incentivo ao encaminhamento para avaliação pré-transplante renal, bem como otimizar a identificação de potenciais doadores, captação e distribuição de órgãos para garantia da equidade no acesso ^2^ ^,^ ^3^.

São limites do estudo a impossibilidade de buscar associação com variáveis clínicas, devido à inexistência de informações nos bancos analisados, assim como ausência de registro de pacientes que foram encaminhados para avaliação pré-transplante renal e tiveram a inscrição em lista de espera negada ou recusaram formalmente o encaminhamento. Logo, torna-se necessário buscar soluções para que os bancos de dados do SUS contenham informações integradas e completas.

## Conclusão

Os resultados do estudo demonstram que o Estado de Minas Gerais apresenta baixo percentual de pacientes inscritos na lista de espera para receber o transplante renal. Destaca-se ainda que não foi observado repercussão positiva na probabilidade acumulada de inscrição dentre os incidentes em diálise nos anos subsequentes a promulgação da *Portaria nº 389/2014*. Percebe-se também disparidades relacionada ao sexo e à idade, uma vez que mulheres e idosos apresentaram menor percentual de inscrição em lista de espera. Foram identificadas iniquidades regionais e relacionadas à natureza jurídica dos centros dialíticos.

Portanto, torna-se primordial que haja esforços direcionados para melhoria nos indicadores pré-transplante renal, com envolvimento do Ministério da Saúde, Secretaria Estadual de Saúde, centros transplantadores e centros dialíticos, a fim de que haja acompanhamento dos resultados e suporte às equipes assistenciais para alcance das metas. Assim como uma análise das disparidades regionais poderão contribuir para a criação de ferramentas de aprimoramento de acordo com a realidade e especificidade de cada localidade.

Sugere-se ainda que ocorra capacitação para gestores e profissionais que atuam na nefrologia, com o intuito de incentivar a inscrição em lista de espera como um indicador assistencial, bem como a inclusão de informações relativas aos encaminhamentos para avaliação pré-transplante renal e inscrição em lista de espera dos pacientes incidentes em diálise nos censos anuais da SBN.
